# Group psychedelic therapy: empirical estimates of cost-savings and improved access

**DOI:** 10.3389/fpsyt.2023.1293243

**Published:** 2023-12-06

**Authors:** Elliot Marseille, Christopher S. Stauffer, Manish Agrawal, Paul Thambi, Kimberly Roddy, Michael Mithoefer, Stefano M. Bertozzi, James G. Kahn

**Affiliations:** ^1^School of Public Health, University of California, Berkeley, Berkeley, CA, United States; ^2^Department of Psychiatry, Oregon Health and Science University, Portland, OR, United States; ^3^Sunstone Therapies, Rockville, MD, United States; ^4^College of Medicine, Department of Psychiatry and Behavioral Sciences, Medical University of South Carolina, Charleston, SC, United States; ^5^Institute for Health Policy Sciences, School of Medicine, University of California, San Francisco, San Francisco, CA, United States

**Keywords:** psychedelic-assisted therapy, MDMA, psilocybin, economics, cost, access

## Abstract

**Objective:**

To compare group and individual psychedelic-assisted therapy in terms of clinician time, costs and patient access.

**Methods:**

Using 2023 data from two group therapy trial sites, one using 3,4-Methylenedioxymethamphetamine (MDMA) to treat posttraumatic stress disorder (PTSD), and one using psilocybin to treat major depressive disorder (MDD), we compared overall variable costs, clinician costs and clinician time required by therapy protocols utilizing groups versus individual patient therapy. Using published literature, we estimated the prevalence of adults with PTSD and MDD eligible for treatment with psychedelic therapy and projected the savings in time and cost required to treat these prevalent cases.

**Results:**

Group therapy saved 50.9% of clinician costs for MDMA-PTSD and 34.7% for psilocybin-MDD, or $3,467 and $981 per patient, respectively. To treat all eligible PTSD and MDD patients in the U.S. in 10 years with group therapy, 6,711 fewer full-time equivalent (FTE) clinicians for MDMA-PTSD and 1,159 fewer for FTE clinicians for psilocybin-MDD would be needed, saving up to $10.3 billion and $2.0 billion respectively, discounted at 3% annually.

**Conclusion:**

Adopting group therapy protocols where feasible would significantly reduce the cost of psychedelic-assisted therapies. By enhancing the number of patients served per clinician, group therapy could also ameliorate the anticipated shortage of appropriately trained clinicians, thereby accelerating access to these promising new therapies.

## Introduction

Increasing evidence from clinical trials suggests that psychedelic therapies may provide breakthrough solutions for difficult-to-treat psychiatric disorders (1–9). These results, coupled with increasing public awareness and advocacy, have fostered a resurgence of interest and a shift in societal attitudes toward these substances. This renewed focus has attracted philanthropic and commercial investment, enabling yet further research, expanded clinical trials, and the development of innovative pharmaceutical approaches (10, 11). The United States FDA has granted Breakthrough Therapy status to 3,4-Methylenedioxymethamphetamine (MDMA) therapy for posttraumatic stress disorder (PTSD) and psilocybin therapy for both major depressive disorder (MDD) and treatment-resistant depression (12, 13). An expanded access program for MDMA therapy for PTSD is ongoing (14). Several local jurisdictions have recently decriminalized psychedelic use or legalized supervised psychedelic administration in the United States (15). While it is too early to be certain, psychedelic therapy may offer transformative treatments that address underlying causes of psychiatric disorders, rather than just manage symptoms.

Despite this momentum, for psychedelic therapies to be widely and equitably accessed, a variety of service delivery issues must be resolved. These include cost-effectiveness, perceived affordability for payers, barriers inhibiting access by underserved populations, and the availability of appropriately-trained clinicians. A strategy widely viewed as an important part of the response to these concerns is the substitution of typical treatment protocols that serve one patient in each session, with protocols that include at least some sessions with two or more patients. Group sessions would, all else equal, lower cost, could have important implications for cost and cost-effectiveness, and for the willingness of third-party payers to sponsor psychedelic-assisted therapies. Another potential benefit of group modalities is that, until the psychedelic therapy workforce is more diversified, specialized groups may provide safer clinical spaces for members of underrepresented populations to undergo identity-affirming psychedelic treatment. By increasing the number of patients who can be treated annually by a full-time equivalent (FTE) therapist, the number of patients able to access qualified clinicians increases correspondingly. Finally, with group models utilizing therapist “teams,” it is possible to employ some practitioners who require lower wages such as properly trained and supervised peers (16).

Most contemporary psychedelic therapy clinical trials utilize two clinicians and one patient in each session. This configuration was chosen to guarantee high levels of safety and to signal to regulators that trial sponsors were focused on safety. However, there is no evidence that this level of clinician intensity is needed for either safety or efficacy. Moreover, research has demonstrated that the efficacy of group therapy is at least equal to that of individual therapy for several indications (17). Irvin Yalom identified eleven evidence-based therapeutic factors of group therapy (18). For example, ‘interpersonal learning’ allows group members to engage with peers to gain greater self-understanding; ‘universality’ allows individuals with similar issues to normalize their symptoms and reduce shame and stigma; and group therapy “instills hope” by allowing participants to witness the progress of others and feel more optimistic about their own treatment.

In the United States, psychedelic research from the 1950’s to 1980’s explored group treatment models with both classic psychedelics (19) and MDMA (20). Early studies with LSD and psilocybin were methodologically heterogeneous: some employed non-directive group administration, and others involved individual psychedelic sessions within a course of group treatment (19). Early group work with MDMA was modeled after indigenous healing ceremonies as well as the work of Stan and Christina Grof (21, 22) and typically involved three therapists with 8–15 patients.

The first 21st century clinical trial to explore psychedelic group therapy using modern research standards was an open-label pilot study with long-term AIDS survivors (*n* = 18) that employed group therapy with group cohorts of six before and after each participant had a traditional individual psilocybin session (23). The study demonstrated the safety and feasibility of group preparation and integration in a psilocybin therapy protocol as well as meaningful improvements in demoralization, complicated grief, and PTSD. The first modern study to look at 1:1 therapy during the psychedelic session built on the previous study and was with patients diagnosed with cancer and MDD (*n* = 30). It used a group approach for one of the two preparatory sessions and for both of the integration sessions after simultaneous administration of psilocybin to three or four participants (9). Participants showed a significant improvement in scales measuring depression. No clinical trials adhering to modern-day scientific rigor have yet examined group therapy with MDMA.

This is the first effort we are aware of to quantify the potential savings associated with the adoption of psychedelic therapy protocols that feature group sessions, and the effect of such protocols on clinician capacity and patient access.

## Methods

### Overview

We obtained empirical data on the costs of psychedelic therapy from two trial sites that incorporate two different protocols, both of which include group therapy features: One is a trial of MDMA-assisted group therapy for veterans with PTSD being carried out by the Social Neuroscience & Psychotherapy (SNaP) Lab housed at the Veterans Administration (VA) Portland Health Care System, funded by the Steven & Alexandra Cohen Foundation, and with study drug provided by the Multidisciplinary Association for Psychedelic Studies (MAPS) Public Benefit Corporation. The second is a completed trial of psilocybin group therapy for the treatment of patients with cancer and MDD conducted by Sunstone Therapies with funding and study drug from Compass Pathways. In addition to the different psychedelic materials (MDMA versus psilocybin), psychiatric indications (PTSD versus MDD), and patient types (veterans versus cancer patients), the protocols differ by the number of sessions and the details of their respective approaches to extending clinician capacity. In both cases, clinicians’ time and the costs required to treat one patient using the group approach is compared with the time and cost needed to treat one patient in an equivalent protocol with one patient in each therapy session. We calculated the savings from substituting the group version for the individual version of each protocol. In addition, we estimated the percentage savings in overall variable costs from using the group version over a range of plausible prices for the psychedelic materials, MDMA for SNaP Lab and psilocybin for Sunstone Therapies. Finally, based on rough projections of the number of adults in the U.S. with PTSD and MDD who would be eligible for MDMA or psilocybin therapy, respectively, we calculated how many years would be required to provide one round of psychedelic therapy to the entire prevalent population assuming varying numbers of FTE clinicians.

### Description of the protocols

Service delivery costs were obtained from two psychedelic therapy trial sites: SNaP Lab’s MDMA-Assisted Group Therapy for the Treatment of Veterans with PTSD (SNaP Lab); and Sunstone Therapies’ Psilocybin Therapy for Cancer Patients with Major Depression (Sunstone).

- SNaP Lab’s protocol consists of four 90-min preparatory sessions; two 8-h open-label MDMA sessions; and eight 90-min integration sessions (four following each of the MDMA sessions). For every patient, one of two doctoral-level psychologists is paired with either a nurse practitioner or a veteran peer counselor. This pair of clinicians is present at each of the three individual sessions, namely: the third preparatory session, the first MDMA session, and the first integration session. The remaining 11 sessions are conducted in a group format with a cohort of six patients. Nine of these group sessions are led by the two psychologists. All four clinicians are present in the remaining two group sessions: the second MDMA session and the first group integration session following that group MDMA session. A psychiatrist supervisor provides support on an as-needed basis.
- Sunstone Therapies’ protocol consists of five sessions, two 90-min preparatory, one 8-h administration of open-label psilocybin, and two 90-min integration sessions. Each of these sessions includes only one patient and one Master’s-level therapist in the treatment room, with the exception of the first preparatory session in which the MD clinical supervisor is also present. For the other four sessions, the “group” aspect consists of the clinical supervisor monitoring, via close-circuit TV, 3–4 patients at a time, each of whom is in a separate treatment room with their individual therapist. The supervisor is thus able to join the primary therapist to support a patient as needed. In this way, the supervising clinician is able to support a group of three – four patients at a time.

See Supplementary Tables 1A,B for a summary of the structure of the protocols.

### Comparative costs

We used a standard micro-costing approach to estimate the variable unit costs of the two protocols (24). Personnel costs were estimated by multiplying the hourly wage plus fringe benefits of each clinician by the hours required to treat each patient per protocol. We adjusted the hours spent on direct service delivery by 20% to reflect the variable cost of billing, scheduling, charting and other indirect activities (see Supplementary material for details). The costs of initial intake, screening, and drug screening were assigned values corresponding to the Medicare reimbursement amounts for the relevant CPT codes. Laboratory costs were derived from charges for each test from Quest^®^ labs. Per protocol, for SNaP Lab we assumed two MDMA sessions with one dose of 120 milligrams and a supplemental dose of 60 milligrams at 1.5 to 2 h for each session, or 360 milligrams of MDMA total per patient. The patients receiving the Sunstone protocol were given one dose of 25 milligrams of psilocybin per patient. The same dosing pattern was assumed in the individual and group formats for both SNaPLab and Sunstone. The expected prices of MDMA and psilocybin are unknown as neither medicines have received FDA approval. However, we applied informed estimates of the likely range of eventual prices and explored the implications of various price points in sensitivity analyses. We derived a total variable cost for each patient completing the protocol by summing per-patient clinician compensation, variable indirect costs, intake, screening and laboratory costs, and the cost of the psychedelic materials themselves. Finally, we repeated the exercise above for SNaP Lab, but assuming that only one patient was seen in all of the sessions along with two therapists; and for Sunstone by eliminating the group monitoring feature and assuming that the supervising therapist was present in all sessions. See Supplementary Table 2 for details on costs.

### Methods for estimating prevalent cases of PTSD and MDD

Our approach to estimating the prevalent cases of PTSD in the United States that are eligible for MDMA treatment according to the protocols used in the MAPS Phase 3 clinical trials is described elsewhere (25). It consists of applying the prevalence of PTSD to the population of U.S. adults and adjusting by the percent that are severe and chronic (50%) and further adjusting by the 22% that are likely to have disqualifying co-morbidities. To estimate the prevalent cases of MDD we took the portion who receive treatment (71.0%) of the 14.8 million adults in the U.S. who had one or more MDD episodes in the past year (26, 27). We applied 30% to this number to reflect the portion who failed treatment or relapsed (28). We then removed an additional 26% to reflect disqualifying co-morbidities for psilocybin-assisted therapies for MDD. See Supplementary material.

### Methods for projecting the effective increase in the clinician workforce from adopting group therapy

For both the group and the individual patient versions, we calculated the number of clinician-hours needed per patient who completes the SNaP Lab or Sunstone protocols. We then divided the number of annual work-hours by this number to derive the number of patients treated per FTE clinician for group versus individual protocols for both SNaP Lab and Sunstone. By dividing the prevalent psychedelic treatment-eligible cases of PTSD and MDD by the number of patients treated per year per FTE clinician, we estimated the number of FTE clinicians that would be needed each year to treat all prevalent cases once over 10 years; 10% per year. We performed a similar calculation to derive the number of years that would be needed to treat each case if 5,000 FTE clinicians were available each year. By multiplying the number of patients treated over 10 years, assuming 10% are treated each year, by the clinician cost per patient, and discounting at 3% annually, we estimated the clinician costs for group and individual patient versions of both the SNaP Lab and the Sunstone protocols. We then repeated this process considering all variable costs, rather than clinician compensation only, for two different price points for the psychedelic materials: $25 and $5 per milligram ($9,000 and $1,800) per treated patient (both MDMA sessions) for MDMA; and $1,500 and $500 per treated patient for psilocybin. In projecting the number of cases that could be treated per year over multiple future years, we simplified by excluding incident cases and deaths.

### Sensitivity and scenario analyses

We present key results using varying assumptions about average group size, the cost of MDMA for the SNaP Lab protocol; and the number of patients simultaneously monitored and the cost of psilocybin for the Sunstone protocol.

## Results

The group protocols entail fewer therapist-hours per patient and thus reduce clinician compensation per patient. For SNaP Lab’s MDMA/PTSD protocol, the savings using base-case values for both the number of patients in group sessions and the cost of MDMA are 36.2 clinician-hours and $3,467 per patient treated, a reduction in clinician costs of 50.9% and of overall variable costs of 20.7%. Group monitoring in Sunstone’s psilocybin/MDD protocol using base case values saves 9.4 clinician-hours and $981 in compensation per patient treated, a reduction in clinician costs of 34.7% and of overall variable costs of 19.0% (see Table 1).

**Table 1:** Comparing group and equivalent individual-patient versions of SNaP Lab (MDMA/PTSD) and Sunstone.

	Group regimen		Equivalent individual regimen		Difference (savings from group regimens)	
	SNaP Lab: MDMA for PTSD					
Clinician type	Therapist-hours	Cost	Therapist-hours	Cost	Therapist-hours	Cost
Supervising therapist	1.0	$215	2.0	$444	(1.0)	($229)
Primary therapist (psychologist)	18.7	$1,962	34.0	$3,574	(15.3)	($1,612)
Adjunct therapist (NP)	7.1	$899	17.0	$2,156	(9.9)	($1,258)
Adjunct therapist (Peer specialist)	7.1	$262	17.0	$630	(9.9)	($367)
	**33.8**	**$3,338**	**70.0**	**$6,804**	**(36.2)**	**($3,467)**
			**Reduction in variable costs**		**20.7%**	
			**Reduction in clinician costs**		**50.9%**	
	Sunstone therapies: psilocybin for major depression					
Lead therapist	4.6	$484	14.0	$1,466	(9.4)	($981)
Regular therapist	14.0	$1,194	14.0	$1,194	0.0	$0
On-call MD	1.6	$167	1.6	$167	0.0	$0
	**20.2**	**$1,846**	**29.6**	**$2,827**	**(9.4)**	**($981)**
			**Reduction in variable costs**		**19.0%**	
			**Reduction in clinician costs**		**34.7%**	

Therapies (Psilocybin/MDD) protocols: Per-patient clinician time, clinician cost, and savings from group protocols as a percent of total clinician costs and of total variable costs. Variable cost reductions assume MDMA costs $9,000, and psilocybin costs $1,500 per patient.

**Figure 1:**
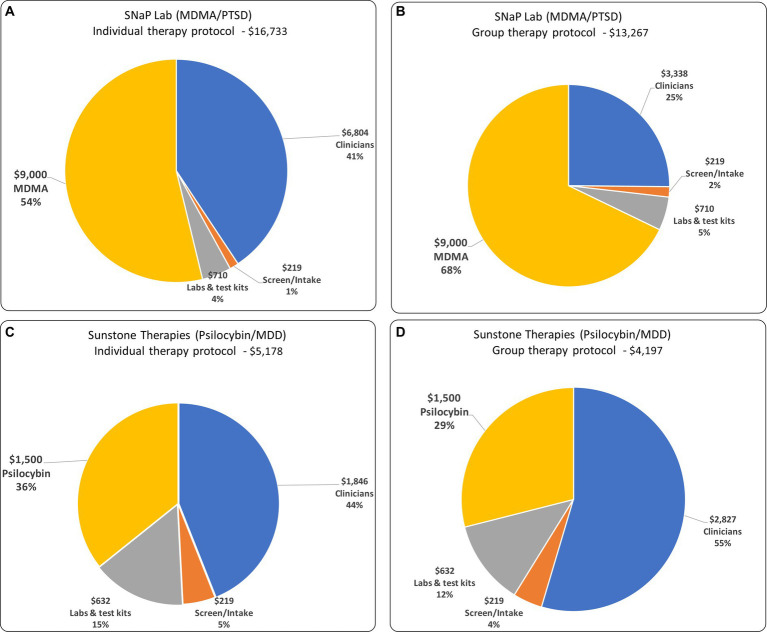
The composition of variable costs and total variable costs for: **(A)** individual therapy protocol for MDD/PTSD; **(B)** group therapy protocol for MDD/PTSD; **(C)** individual therapy protocol for Psilocybin/MDD; and **(D)** group therapy protocol for Psilocybin/MDD.

In both the group and individual patient versions of each protocol, and assuming MDMA and psilocybin costs of $9,000 and $1,500 per patient respectively, psychedelic medications and clinician compensation constitute the largest cost components. For SNaP Lab, they are 68 and 25%, respectively, of the total. For the equivalent individual-patient protocol, MDMA and clinician compensation constitute 54 and 41%, respectively. For Sunstone’s psilocybin/MDD protocol, the psilocybin and clinician compensation constitute 36 and 44% of the total variable cost, respectively. For the equivalent individual-patient protocols psilocybin and clinician costs constitute 29 and 55%, respectively (see Figure 1).

### Sensitivity analyses

**Figure 2:**
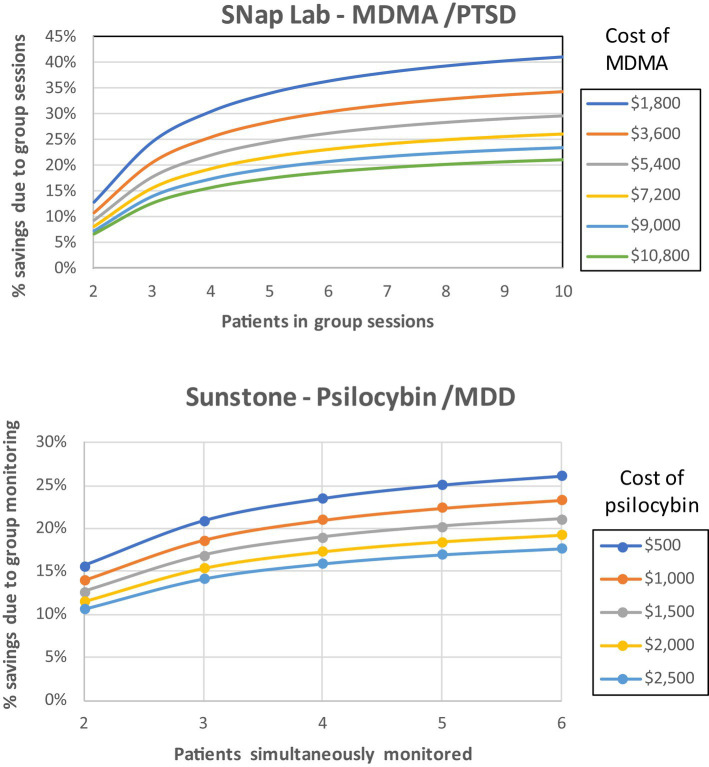
Percent reduction in variable cost due to group therapy, by group size, and per-patient cost of psychedelic material. Base-case values for medication costs are $9,000 per patient (2 doses of $120 mg plus 2 supplemental dose of 60 mg) for MDMA (SNaP Lab); and $1,500 per patient (1 dose of 25 mg) of psilocybin (Sunstone); and six patients per group session in SNaP Lab’s protocol and four patients monitored simultaneously in the Sunstone protocol.

For both SNaP Lab and Sunstone, the relative savings in variable costs from the group approaches are sensitive to the cost of the medications and the number of patients per group. If MDMA costs in SNaP Lab were $1,800 rather than the base-case value of $9,000, savings from group therapy would be 36.4% rather than 20.7%, assuming that six patients are treated per group sessions. If psilocybin costs in Sunstone’s protocol were $500, rather than the base-case value of $1,500, savings would be 23.5% rather than 19.0% assuming that four patients are simultaneously monitored. If increasing groups size beyond current protocols were found to be safe and effective, the incremental reduction in costs would be modest. For example, increasing group size from 6 to 8 patients in SNaP Lab’s MDMA/PTSD protocol would increase the reduction in variable costs from 20.7 to 22.4% assuming the base-case MDMA cost of $9,000, an additional savings of $277 per patient (see Figure 2).

For the SNaP Lab and the Sunstone protocols, group therapy would allow an additional 28.9 and 29.5 patients, respectively, to be served annually per FTE clinician. For SNaP Lab, 6,711 fewer FTE clinicians would be required each year to treat each prevalent eligible patient with PTSD in the U.S within 10 years (10% per year). For Sunstone, 1,159 fewer FTE clinicians would be required each year to treat prevalent eligible patients with MDD within 10 years. Compared with individual therapy, 5,000 FTE clinicians providing group therapy exclusively using the SNaP Lab protocol could treat prevalent cases in 13.4 fewer years. Using Sunstone’s group therapy model would reduce the time needed for 5,000 FTE clinicians to treat prevalent MDD cases by 2.3 years (see Table 2).

**Table 2:** SNaP Lab: therapists and therapist costs to treat prevalent severe/extreme, treatment-resistant PTSD cases in 10 years.

SNaP Lab: MDMA / PTSD				
	Individual	Group	Absolute savings	Relative savings
Patients treated per year per FTE therapist	27.0	55.9	28.9	51.7%
FTE therapists needed to treat prevalent cases in 10 years	12,977	6,266	6,711	
Years to treat prevalent cases if 5,000 FTE therapists	26.0	12.5	13.4	
Clinician cost^1^ to clear backlog in 10 years	$20.3 Billion	$10.0 Billion	$10.3 Billion	
Total variable cost^1^, MDMA $9,000 per patient	$50.0 Billion	$39.6 Billion		20.7%
Total variable cost^1^, MDMA $1,800 per patient	$28.5 Billion	$18.1 Billion		36.4%
Sunstone therapies: psilocybin/MDD				
Patients treated per year per FTE therapist	63.8	93.3	29.5	46.2%
FTE therapists needed to treat prevalent cases in 10 years	3,660	2,501	1,159	31.7%
Years to treat prevalent cases if 5,000 FTE therapists	7.3	5.0	2.3	31.7%
Clinician cost^1^ to clear backlog in 10 years	$5.6 Billion	$3.7 Billion	$2.0 Billion	34.7%
Total variable cost^1^, psilocybin $1,500 per dose	$10.3 Billion	$8.4 Billion		19.0%
Total variable cost^1^, psilocybin $500 per dose	$8.3 Billion	$6.4 Billion		23.5%

^1^Costs discounted at 3% annually.

Sunstone: therapists and therapist costs to treat prevalent MDD cases in 10 years. Costs discounted at 3% annually.

## Discussion

We found that group therapy can reduce psychedelic-assisted therapies costs and expand the effective supply of trained clinicians. This has important implications for wide and equitable access since, following FDA approval, the demand for psychedelic therapies may outstrip the supply. Average wait times for behavioral health interventions are already weeks or months, and the demand for mental health services has surged in recent years (29). The additional demand for psychedelic therapies may exacerbate the problem and could crowd out access to conventional therapies. The supply of trained clinicians to meet this demand is likely to be constrained. According to the Administrator of the Substance Abuse and Mental Health Services Administration, there will be a shortage of 31,000 mental health practitioners by 2025 (30). An April, 2023 Harris Poll, found that 48% of mental health practitioners stated that workforce shortages caused them to consider alternative employment (30). Potential psychedelic therapists may be discouraged by tuition costs, a shortage of training slots, time constraints, and the uncertain regulatory environment (31). Thus, other responses to these issues, in addition to group therapy should be considered. Among these are providing greater incentives for people to enter careers in mental health, and allowing nurses and other non-MD or PhD-level staff to administer psychedelic therapy (32).

A key finding of this study is that the adoption of psychedelic group therapy models may dramatically increase the number of patients who can be treated per FTE clinician. In the context of a constrained supply of trained therapists this has important implications for enhancing access. Because the prices of psilocybin and especially of MDMA are likely to constitute large portion of total costs, relative (but not absolute) cost reductions from group therapy will depend importantly on prices of MDMA and psilocybin following FDA approval. Group therapy requires specialized skills from practitioners. Quality should not be compromised in the pursuit of cost containment.

### Limitations of this analysis

This analysis examines cost differences and does not account for potential differences in benefits between group and individual therapy protocols. In addition, the projection of clinicians required to treat prevalent cases over time is approximate, as it ignores incident cases and mortality. Due primarily to the uncertainty in the price of the psychedelic medications, relative reductions in overall variable costs are uncertain. We restricted our cost evaluation to variable costs, i.e., those that are a direct function of the number of patients served. We excluded fixed overhead and administrative costs, potential additional training that clinicians might need, as well as refurbishment costs that may be entailed in preparing a site for the number and size of treatment rooms required by a change to group therapy. We assume that psychedelic therapy with some group sessions is as safe and effective as individual therapy. No randomized trials have compared them directly, but a review found no difference in efficacy between group therapy and control conditions (19).

It is unknown what portion of people who are willing to undergo the rigors of psychedelic therapies will be amenable to group therapy. Group therapy should therefore be an option, not a requirement. While some patients find comfort in shared experience, others may prefer more privacy. The Sunstone model of separate rooms with shared oversight via TV is an alternative. It combines substantial reductions of personnel time and costs, while preserving the privacy of individual therapy.

This study examined two group models for group psychedelic-assisted treatment, but there may be other efficacious and cost-saving models. Different group sizes, number of sessions, and clinician-to-patient ratios could be explored. However, incremental savings decline with incremental increases in group size. In addition, there is a point in the reduction of clinician-to-patient ratios where safety considerations inhibit further decreases. These thresholds likely vary by psychiatric indication and psychedelic compounds used: PTSD patients may require smaller groups than patients with a cancer diagnosis and an anxiety disorder. MDMA, because its effects are more predictable than psilocybin, may be more conducive to larger groups. Establishing these thresholds is an important area of research.

Savings could be greater in other proven MDMA-assisted therapy protocols for PTSD. The only MDMA-assisted therapy protocol for which Phase 3 trials have been conducted, entailed three MDMA sessions (1), rather than the two sessions of the SNaP Lab’s protocol. Earlier analysis by our research group found that the addition of the third session was cost-effective compared with two MDMA sessions (33). Thus, if the three-MDMA-session model became standard psychedelic therapy for PTSD, the importance of group sessions in containing costs and extending the clinician workforce would be even more pronounced.

### Public health implications

Group therapy reduces clinician costs by 50.9% for SNaP Lab MDMA/PTSD protocol and by 34.7% for the Sunstone’s psilocybin/MDD protocol. Thus, group therapy, where it can be applied, can double the effective supply of psychedelic therapy clinicians. The time required to reach a target population of patients is correspondingly halved.

The effect of adopting group models on overall variable cost reduction is more modest, 20.7% for MDMA/PTSD and 19.0% for psilocybin/MDD, using reasonable estimates of the cost of the psychedelic medications. Even these limited reductions would enhance cost-effectiveness and perceived affordability by insurers and thus increase the likelihood of wide adoption.

## Data availability statement

The raw data supporting the conclusions of this article will be made available by the authors, without undue reservation.

## Author contributions

EM: Conceptualization, Formal analysis, Methodology, Project administration, Supervision, Validation, Writing – original draft, Writing – review & editing. CS: Conceptualization, Formal analysis, Methodology, Writing – original draft, Writing – review & editing. MA: Conceptualization, Methodology, Writing – original draft, Writing – review & editing. PT: Conceptualization, Data curation, Formal analysis, Methodology, Writing – review & editing. KR: Writing – review & editing, Conceptualization. MM: Writing – review & editing. SB: Conceptualization, Formal analysis, Methodology, Writing – review & editing. JK: Conceptualization, Formal analysis, Methodology, Validation, Visualization, Writing – original draft, Writing – review & editing.
