# Supplementary material for: Group psychedelic therapy: empirical estimates of cost-savings and improved access
Source: Front Psychiatry. 2023 Dec 6;14:1293243. doi: 10.3389/fpsyt.2023.1293243 (PMC10731307; doi:10.3389/fpsyt.2023.1293243)
Supplement: Supplementary file 1 [file Table_1.DOCX]

**Supplementary Material**

**Specification of the SNaP Lab protocol for treating PTSD with MDMA- assisted therapy and Sunstone Therapies protocol for treating major depression with psilocybin-assisted therapy.**

**Supplementary table 1.** Summary of the SNaP Lab protocol showing the time in minutes of each session, the number of patients in each, and the distribution of clinician time across sessions.

**Supplementary table 2.** Summary of the Sunstone Therapy protocol showing the time in minutes of each session, the number of patients in each, and the distribution of clinician time across sessions.
